# FBXW7 Directly Ubiquitinates and Degrades CTNNB1 Mediating the Suppression of ENKUR in Endometrial Cancer

**DOI:** 10.7150/ijbs.104067

**Published:** 2025-02-10

**Authors:** YaHui Liu, Qian Wang, QiRun Guo, Ying Zhu, Li Lin, ChunYan Yang, Bin Gong, Weiwei Yan, RenTao Hou, Yao Tang, XiuQiong Wu, Xinhui Liu, BeiXian Zhou, WeiYi Fang, LuYun Shu, SuiQun Guo

**Affiliations:** 1 Oncology department, Southern Medical University Hospital of Integrated Traditional Chinese and Western Medicine, Southern Medical University, Guangzhou 510315, China.; 2 Department of Obstetrics and Gynecology, The Third Affiliated Hospital/The Third Clinical Medicine School of Southern Medical University, Guangzhou 510315, China.; 3 Department of Gynecology and Oncology, Huaihua Cancer Hospital, Huaihua 418100, China.; 4 Department of radiotherapy, Shunde Hospital, Southern Medical University (The First People's Hospital of Shunde Foshan), 1# Jiazi Road, Foshan, 528300, Guangdong, China.; 5 Department of Gastrointestinal Oncology, Shandong Cancer Hospital and Institute, Shandong First Medical University & Shandong Academy of Medical Science, Jinan 250117, China.; 6 The People's Hospital of Gaozhou, Gaozhou 525200, China.

**Keywords:** ENKUR, FBXW7, CTNNB1, ubiquitination, endometrial cancer

## Abstract

Enkurin (ENKUR) is a tumor suppressor in some malignancies. However, its role in endometrial cancer (EC) remains unknown. Here, we firstly observed that reduced ENKUR expression promotes progression and poor prognosis in EC. Moreover,the overexpression of ENKUR suppressed the proliferation, migration, invasion, and intrahepatic dissemination of EC *in vitro* and *in vivo*. Repressing ENKUR expression by small-interfering RNA significantly reversed the inhibition of cell proliferation and invasion *in vitro*. We used co-immunoprecipitation combined with mass spectral analysis to identify the potential interactive proteins of ENKUR. Based on Gene Ontology analysis, we discovered that Wnt/β-catenin (Wnt/CTNNB1) signaling is a ENKUR-modulated key pathway. ENKUR binds to CTNNB1, significantly repressing its protein expression. Furthermore, ENKUR also binds to E3 ligase F-box and WD repeat domain containing 7 (FBXW7), a critical tumor suppressor. Interestingly, the latter binds to CTNNB1 and S502 of CTNNB1 is the key binding site, thereby increasing its protein ubiquitination and degradation. Finally, we confirmed that the predominant ubiquitination sites of CTNNB1 are located at K281 and K394. Transfection of ENKUR-overexpressing EC cells with CTNNB1 reversed the suppressive effects on tumor growth and invasion. ENKUR may be a tumor suppressor via recruiting FBXW7 to directly ubiquitinate and degrade CTNNB1 in EC.

## Introduction

Endometrial carcinoma (EC) is the most common gynecologic malignancy worldwide. It is classified into different subtypes, with endometrioid and serous carcinoma representing 80-85% and 5-10% of newly diagnosed cases, respectively^1^, ^2^. Despite great progress achieved in the treatment and diagnosis of EC^3^-^8^, the incidence of advanced EC continues to increase^9^. However, the pathogenesis of EC remains to be clarified.

Enkurin, also known as the “enhanced nuclear factor kappa-B receptor” (ENKUR), is located at chromosomal band 10p12.1. It encodes a calmodulin that was originally identified using yeast two-hybrid screening in a study of transient receptor potential canonical (TRPC)^10^. ENKUR is a conserved protein of flagella and cilia^11^. Therefore, ENKUR protein is a necessary protein for sperm cilium movement and transport in the female fallopian tube, playing important roles in signal transduction and gamete fusion during the fertilization process of *Cystoisospora suis*^10^, ^12^-^14^. Additionally, an increase in ENKUR protein has been reported as an early diagnostic biomarker to predict the occurrence of noise-induced hearing loss at a very early stage^15^. At present, there are very few reports on the role of ENKUR in tumors. It has been identified as a novel marker for myeloproliferative neoplasms from platelet, megakaryocyte, and whole blood specimens^16^. Follow-up studies demonstrated that ENKUR is a tumor suppressor participating in the pathogenesis of colorectal cancer (CRC), lung adenocarcinoma (LUAD), hepatocellular carcinoma (HCC) and nasopharyngeal carcinoma (NPC)^17^-^21^. However, thus far, studies have not focused on the role of ENKUR in EC. In the present study, we detected the expression of ENKUR in EC, and evaluated its tumor suppressor role in human EC cells and nude mice.

## Materials and methods

### Tissue specimens

A total of 178 paraffin-embedded EC specimens and 23 endometrial epithelium samples were obtained from The Third Affiliated Hospital of Southern Medical University, Guangzhou City, China. Consent from the patients and approval from the Ethics Committee of the Third Affiliated Hospital of Southern Medical University were obtained before using the clinical samples for research purposes. Subcutaneous and intrahepatic dissemination tumors obtained from each group of nude mice (five mice per group) were immediately embedded in paraffin, sliced (thickness: 0.3µm), and subjected to hematoxylin-and-eosin (HE) and immunohistochemistry (IHC) staining.

### IHC staining

ENKUR and proliferating cell nuclear antigen (PCNA) protein expression levels were detected by IHC staining. Immunochromogenic reagent (UltraSensitive sp mouse/rabbit) (MXB Biotechnologies, Fuzhou, China) and DAB kit (ZSGB BIO) were used for IHC staining. IHC was performed as previously described^22^. Two senior doctors in the dapartment of pathology evaluated the results of IHC staining. IHC scoring was based on a semiquantitative method according to the intensity and percentage of staining, as previously described^22^. The intensity of staining was scored using a scale ranging 0-3 (0=negative staining, 1=weakly positive staining, 2=moderately positive staining, and 3=strongly positive staining). The percentage of staining was estimated based on a scale ranging 0-4 (0=none, 1=positive staining in 1-25% of cancer cells, 2=positive staining in 26-50%; 3=positive staining in 51-75%; and 4=positive staining in 76-100%). The IHC score was determined by multiplying the intensity score by the percentage score; scores ≥6 and <6 denoted high and low expression, respectively. Details of the antibodies used are listed in Table S1.

### Cell culture

RL95-2 and HEC-1-B cell lines obtained from the Cancer Research Institute of Southern Medical University (Guangzhou, China) were cultured in DMEM/MEM (Vivacell, Shanghai, China) supplemented with 1% penicillin-streptomycin (Biological Industries, Israel) and 10% fetal bovine serum (FBS) (Biowest, Nuaillé, France). HEK293T cells were acquired from the research group of Lukui Chen at the Integrated Hospital of Traditional Chinese Medicine of Southern Medical University, and were cultured in DMEM (Vivacell) containing 10% FBS (Biowest). All cells were incubated in 95% air and 5% CO_2_ at 37°C.

### RNA extraction and reverse transcription-quantitative-polymerase chain reaction (RT-qPCR)

Total RNA was isolated from the cell lines using the Total RNA Isolation Kit (Foregene, Chengdu, China). The concentration of total RNA was determined using Nanodrop 2000 (Thermo Fisher Scientific, Guangzhou, China). ENKUR and CTNNB1 mRNA was amplified using SYBR Premix Ex Taq, and reverse transcription was conducted using the PrimeScript RT reagent kit (Takara Biotechnology Co., Ltd., Dalian, China) to quantify ENKUR and CTNNB1 mRNA expression. Reverse transcription was performed at 37°C for 15 min and 85°C for 5 s. Glyceraldehyde-3-phosphate dehydrogenase (GAPDH) and β-actin were used as internal controls for ENKUR and CTNNB1. The PCR primers used in the present study are listed in Table S2. Fold-changes for ENKUR and CTNNB1 mRNA expression levels were calculated using the 2^-ΔΔCt^ method.

### Lentivirus infection and transient transfection

Lentiviral particles carrying human ENKUR short hairpin RNA ENKUR and empty vector controls (PLV-Ctr) (Table S3) were constructed by GeneChem Company (Shanghai, China). RL95-2 and HEC-1-B cells were infected with lentiviral vectors with green fluorescent protein (Fig. S1C)**.** Transient transfection using plasmids or small-interfering RNAs of FBXW7 and CTNNB1 were designed and synthesized by RiboBio Inc. (Guangzhou, China) (Table S3). Plasmids with His or Flag containing CTNNB1 mutant sequences were obtained from Miaoling Biology (Wuhan, China) (G42952, G42950, G42949, G42913, G42914). At 4-5 h prior to transfection, RL95-2, HEC-1-B or 293T cells were seeded into a plate (Nest Biotech, Shanghai, China) at 30-50% confluence. Next, the cells were transfected with small-interfering RNA (siRNA) or plasmids using Lipofectamine™ 3000 (Invitrogen Biotechnology, Guangzhou, China) for the following experiments, according to the instructions provided by the manufacturer.

### Western blotting

Western blotting was performed using the Mini-PROTEAN Tetra- and Mini Trans-Blot (Bio-Rad, Hercules, CA, USA) systems, as previously described (https://www.westernblotprotocol.com).

Images were captured using a Chemiluminescence Imaging System (Minichemi, Beijing, China). We used antibodies against GAPDH, β-tubulin, ENKUR, CTNNB1, FBXW7, β-actin, hemagglutinin (HA), ubiquitin (UB), K48, Flag, and His, which are listed in Table S1.

### MTT assays and colony formation assays

The MTT assay was conducted to detect cell viability according to the instructions provided by the manufacturer (Sigma-Aldrich, Shanghai, China). The optical density value was measured at 490 nm wavelength. EC cells were seeded at a density of 300-800 cells/well for colony formation. After 2 weeks of culture, the cells were washed with phosphate-buffered saline (PBS) and stained with hematoxylin solution. The number of colonies in each well was determined. All experiments were performed at least thrice.

### EdU incorporation

Cells were seeded into 96-well plates at 8,000 cells/well, and experiments were performed in triplicate for each condition in each group. At 6 h after seeding, the culture medium was changed to FBS-free or low-FBS medium for 24 h to synchronize the cell cycle. EdU staining was performed using the Cell-Light EdU Apollo567 *in vitro* Kit (Ribobio Inc., Guangzhou, China) and 4',6-Diamidino-2-phenylindole dihydrochloride (DAPI; 5 μg/mL) (Beyotime Biotechnology, Shanghai, China) according to the instructions provided by the manufacturer.

### Flow cytometric analysis

To assess the cell cycle, a suspension of 1 million cells per milliliter was treated with 70% ethanol and stored at 4°C for a minimum of 12 h. Subsequently, the cells were exposed to RNase A at a concentration of 50 µg/mL at 37°C for 30 min. The DNA within the cells was stained using propidium iodide at a concentration of 50 µg/mL. The cell cycle distribution was determined using a LSRFortessa (BD Biosciences, Franklin Lakes, NJ, USA) flow cytometer equipped with fluorescence-activated cell sorting capabilities.

### Wound-healing assay

EC cells transfected with plasmids, lentiviruses or siRNAs were seeded into six-well plates. We performed artificial wounds on cells using a 100 μL pipette tip, and the cells were subsequently washed. After incubation for 48 h in a humidified chamber, the degree of wound healing was determined to estimate the migratory ability of cells.

### Transwell assay

Cells (1×10^5^ cells/well) were seeded into a 24-well plate and washed twice with PBS. The upper chamber of the Transwell plate contained 100 μL of DMEM culture medium, while the lower chamber contained 500 μL of DMEM with 10% FBS. After culture at 37°C for 18-20 h, the cells that had not penetrated the membrane surface in the upper chamber were wiped off, and the remaining attached cells were washed thrice with PBS. Thereafter, the cells were fixed with paraformaldehyde for 10 min, stained with crystal violet for 15 min, and washed with PBS. Images of cell migration were captured under a microscope (Olympus Corporation, Tokyo, Japan) (100× magnification). Three fields of view were randomly selected to calculate the number of cells, with the average value denoting the number of cells penetrating the membrane.

### Boyden assay

Initially, 24 µg/mL Matrigel (BD Biosciences, Franklin Lakes, NJ, USA) was applied to precoat Transwell membranes (BD Biosciences) for 30 min. Next, a total of 1×10^5^ cells were resuspended in 100 µL of serum-free DMEM culture medium and added to the upper chamber. Subsequently, DMEM culture medium containing 10% FBS was added to the lower chamber (500 µL). The filter was collected 24-30 h later, and staining with crystal violet solution for 3 min was performed. The cell capacity for invasion was assessed based on the average value obtained from three random fields.

### Liquid chromatography-tandem mass spectrometry analysis and Gene Ontology (GO) enrichment analysis

The entire process of library construction and sequencing was performed at Shanghai Lifegenes Technology Co., Ltd. (Shanghai, China). Mass spectrometry analyses were performed at Shanghai Lifegenes Technology Co., Ltd, using 20 µg of proteins from the Co-immunoprecipitation (Co-IP) assays. GO enrichment analysis of differentially expressed genes was conducted using the clusterProfiler R package (v3.18.1)^23^ (https://guangchuangyu.github.io/software/clusterProfiler). GO terms with p-values <0.05 denoted significant enrichment of differentially expressed genes.

### *In vivo* tumorigenesis in nude mice

*In vivo* experiments were approved by the Animal Care and Use Committee of Southern Medical University and were performed in accordance with the National Institute of Health Guide for the Care and Use of Laboratory Animals. Nude mice (age: 4 weeks) were purchased from SPF (Beijing) Biotechnology Co., Ltd. (Beijing, China). The subcutaneous xenograft tumor model was constructed as follows^22^. A total of 5×106 EC cells in logarithmic growth were transfected with the ENKUR-overexpression plasmid or vector in 0.1 mL of Hanks' balanced salt solution (Chemical Book, Shanghai, China). Thereafter, they were subcutaneously injected into the mice (BALB/c; nu/nu; age: 4 weeks; sex: female; weight: 12-13 g; n=5 per group). The long and short diameters of the tumors were measured once every 3 days using a Vernier caliper. The tumor volume was calculated as follows: V=ab^2^/2 (a=tumor length, b=tumor width). At 21 days, the mice were sacrificed, and the tumors were removed, fixed using 4% paraformaldehyde, embedded in paraffin, and sectioned for subsequent detection (i.e., HE staining and PCNA staining)^22^.

A mouse model of intrahepatic dissemination was constructed as follows. Mice (BALB/c; nu/nu; age: 4 weeks; sex: female; weight: 12-13 g; n=5 per group) were divided into four groups (i.e., RL95-2-Vector, RL95-2-ENKUR, HEC-1-B-Vector, and HEC-1-B-ENKUR cells) and anesthetized by intraperitoneal injection of 0.5% pentobarbital sodium (50 mg/kg; Sigma, Shanghai, China). The liver was exposed ,1×10^6^ EC cells with stable ENKUR overexpression and control cells were resuspended in PBS and injected under the liver capsule using a needle, thereafter, the abdominal incision was sutured and disinfected. After 60 days, the mice were sacrificed, and the livers were isolated to observe intrahepatic dissemination tumors under fluorescence microscopy. Next, the intrahepatic dissemination tumors were fixed using 4% paraformaldehyde for observation.

### Co-IP

Co-IP was carried out using a Pierce Co-IP kit (Thermo Scientific, Waltham, MA, USA). Briefly, total protein was extracted and quantified. A total of 1,000 mg of protein in 400 mL of supernatant was incubated with 10 mg of antibodies against ENKUR, CTNNB1, Flag, His, UB, FBXW7, and immunoglobulin G (IgG) for 12 h at 4°C. The beads (Bimake, Shanghai, China) were washed and eluted in sample buffer, and boiled for 5 min at 95°C. Immune complexes were subjected to western blotting analysis. Anti-IgG antibody was used as a negative control.

### Immunofluorescence and confocal microscopy

Cells were separated and seeded on coverslips in a 48-well plate. After adherence, the cells were fixed using paraformaldehyde (4%) and permeabilized with Triton X-100 (0.2%). Subsequently, the cells were incubated with specific antibodies (i.e., against FBXW7, CTNNB1, and ENKUR) and counterstained with DAPI (0.2 mg/mL). Finally, images were captured under a Carl Zeiss LSM800 confocal laser scanning microscope (Zeiss, Germany).

### Cycloheximide (CHX) chase and ubiquitination assays

CHX chase and ubiquitination assays were performed as previously described^19^, ^24^. CHX (50 µg/mL) (S7418; Selleck, Shanghai, China) was used to treat EC cells for various periods of time. Proteins were extracted using radioimmunoprecipitation assay lysis buffer, mixed with sodium dodecyl sulfate-loading buffer, boiled for 10 min, and subjected to immunoblotting. For the ubiquitination assays, cells were treated with MG132 (20 µM) (S2619; Selleck) for 12 h and proteins were harvested. Proteins were extracted using IP lysis buffer, and IP was performed with anti-CTNNB1 antibodies. Harvested proteins were resolved in sodium dodecyl sulfate-loading buffer, boiled for 10 min, and subjected to immunoblotting.

### Protein-protein docking studies

Protein-protein docking was performed using the HDOCK server (http://hdock.phys.hust.edu.cn/). This docking program initially sampled the putative binding modes between the two proteins through a fast Fourier transform-based global search method 7. Thereafter, the sampled binding modes were evaluated with an improved iterative knowledge-based scoring function for protein-protein interactions^25^-^27^. The electron microscopy structures of CTNNB1 (Protein Data Bank [PDB] identifier: 8Y0G) as receptors and the X-ray crystallographic structures of FBXW7 (PDB identifier: 2OVP) were considered ligands for the protein-protein docking. These structures were recovered from the PDB (https://www.rcsb.org/). PyMol software (DeLano Scientific LLC, Shanghai, China) was used to visualize the docking results for the members with the highest scores.

### Statistical analysis

Statistical analyses were performed with the SPSS 19.0 statistical software package (IBM Corp., Armonk, NY, USA). Data are expressed as the mean±standard deviation from at least three independent experiments. Comparisons were performed using a Student's *t*-test for two groups, one-way analysis of variance (ANOVA) for multiple groups, and a parametric generalized linear model with random effects for tumor growth. Survival analysis was performed using the Kaplan-Meier method. All statistical tests were two-sided. The p-values <0.05 indicated statistically significant differences.

## Results

### Reduced ENKUR promotes EC progression

**Figure 1:**
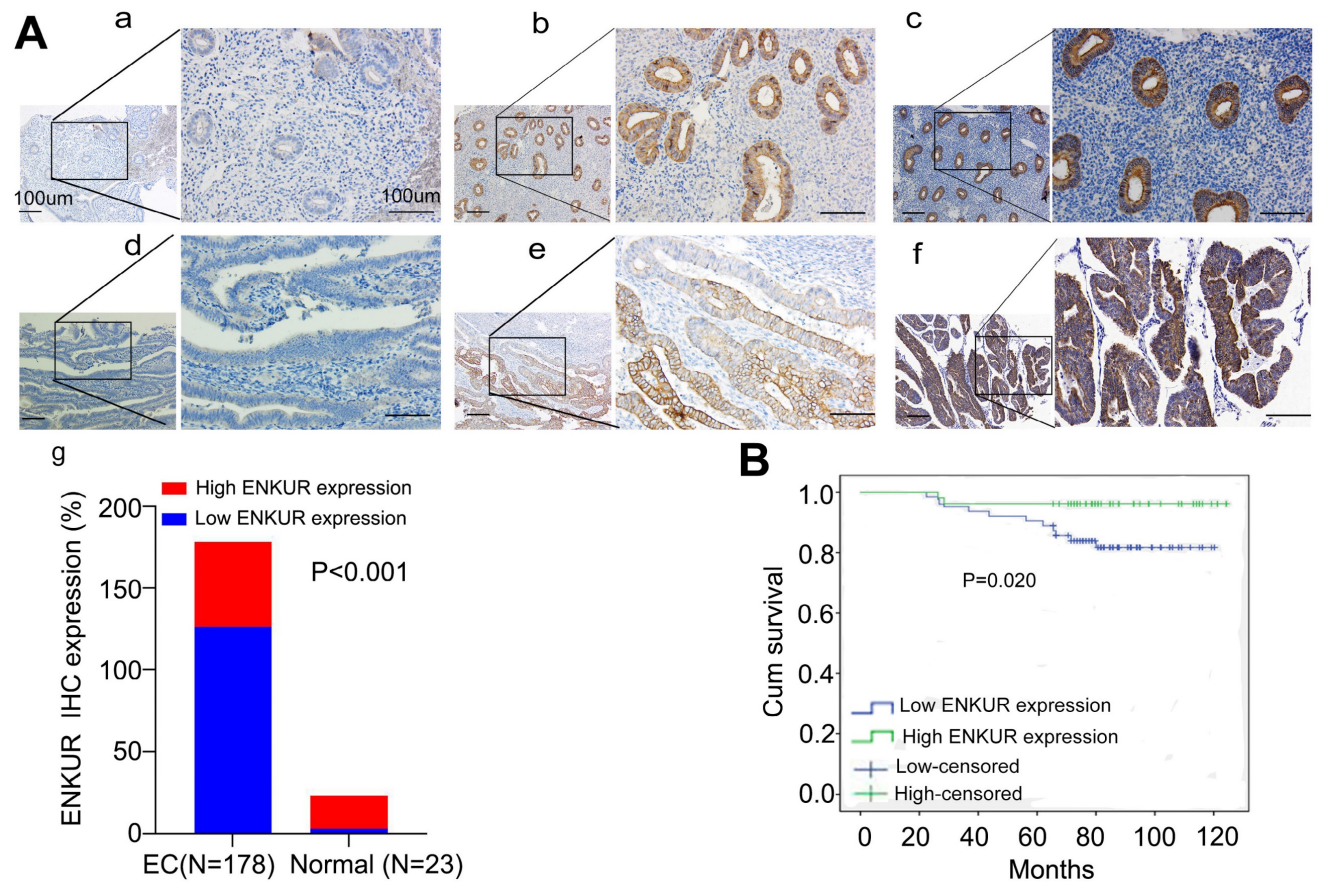
Lower expression of ENKUR promotes EC and is associated with a worse prognosis. (A) IHC staining of ENKUR in EC tissues and normal endometrial tissues. a: negative of ENKUR in normal endometrial tissues; b: positive of ENKUR in normal endometrial tissues; c: strongly positive of ENKUR in normal endometrial tissues; d: negative of ENKUR in EC samples; e: positive of ENKUR in EC tissues; f: strongly positive of ENKUR in EC tissues; g: a total 29.2% of endometrial cancer samples while 85% of normal endometrial tissues were strongly positive for ENKUR, ENKUR was significantly lower expressed in endometrial cancer compared with that in endometrial tissues (P<0.001). (B) A Kaplan-Meier survival analysis is shown for the overall survival of 178 EC patients on the basis of ENKUR expression. A log-rank test was used to calculate P-values, P=0.020. Scale bars, 100μm.

We initially sought to investigate whether ENKUR is involved in the pathogenesis of EC. Thus, we used the online Gene Expression Profiling Interactive Analysis (GEPIA) and UALCAN databases to predict changes in ENKUR expression. The data showed that both ENKUR mRNA and protein expression levels were upregulated in EC tissues compared with healthy endometrial and endometrial hyperplasia samples (Fig. S1A, B). Interestingly, we observed that high *ENKUR* expression predicts a favorable prognosis for patients with EC (Fig. S1C). We further confirmed that ENKUR expression is significantly downregulated in EC tissues compared with normal endometrial tissues by IHC (Fig. 1A and Table 1). The clinical characteristics of patients with EC are summarized in Table 2. There were no significant associations found between ENKUR expression and patient age, clinical stage, hormone levels, hypertension history, etc. Nevertheless, we observed that low ENKUR expression was associated with shorter patient survival than high ENKUR expression (log-rank test, p=0.020) (Fig. 1B). This evidence was consistent with the survival data obtained from the database analysis.

### ENKUR overexpression decreases EC cell growth *in vitro* and *in vivo*

**Figure 2:**
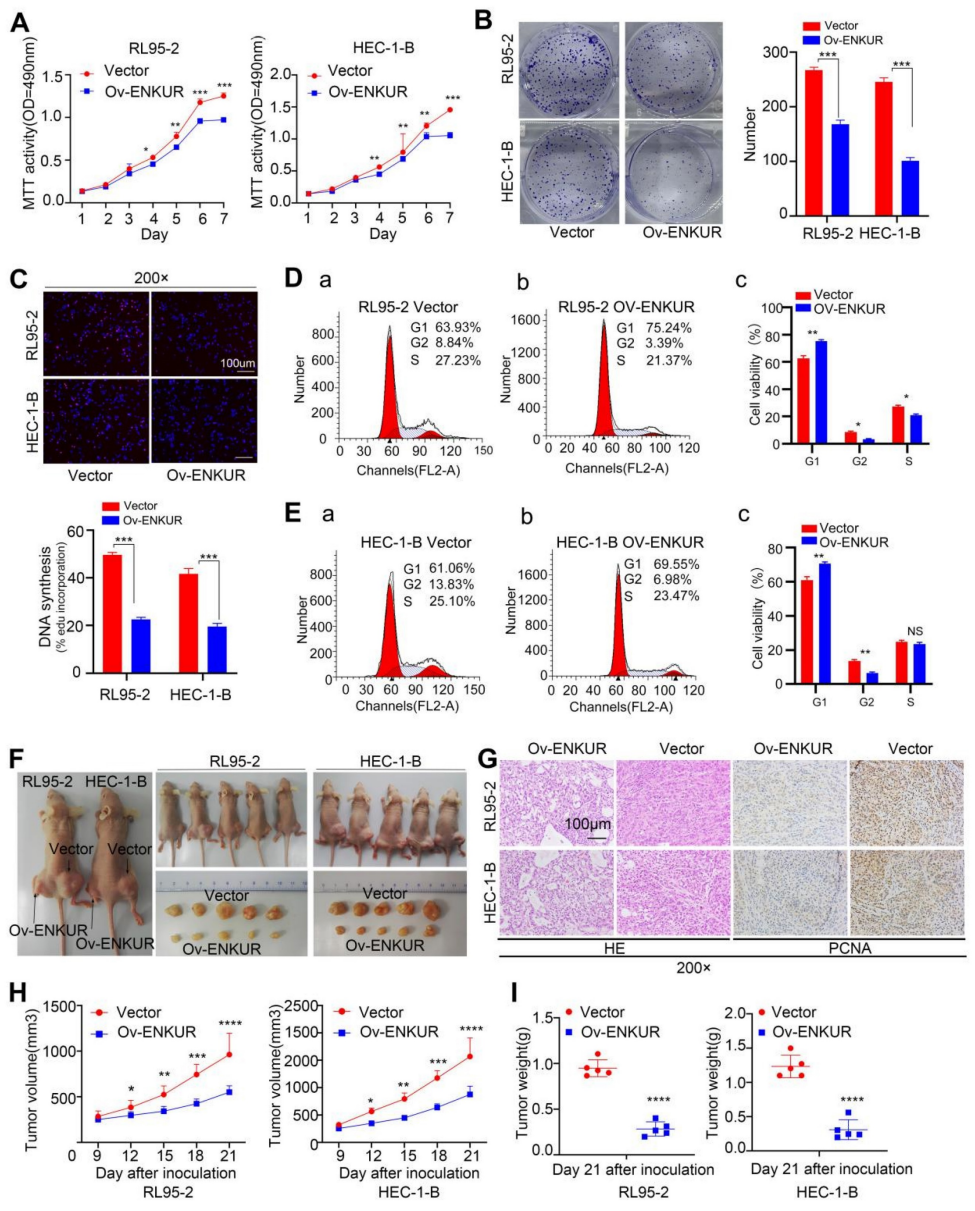
ENKUR reduces EC proliferation in vitro and *in vivo* (Stable interference). The effects of ENKUR on the cell proliferation of RL95-2 and HEC-1-B cells were examined by (A) MTT assay, (B)Colony formation assay, (C) EdU experiments and (F) Xenograft tumour model in nude mice. (D-E) Flow cytometry analysis of EC cells showing G1 arrest of cell cycle. (F) Xenograft tumour model in nude mice. (G) Xenografts were stained with HE and subjected to immunohistochemistry for pcna expression (n=5 per group). (H)Tumor volume of xenografts in nude mice were measured (n=5 per group). (I)Tumor weights of xenografts in nude mice were measured (n=5 per group). Student's t test. Mean±SD. **p < 0.01, ***p < 0.001, ****p < 0.0001. Scale bars, 100µm.

We also sought to verify the influence of ENKUR in EC cell proliferation. For this purpose, we used lentiviruses to establish stable ENKUR expression in EC cells (Fig. S1D). Significant ENKUR overexpression was confirmed in both cell lines (Fig. S1D, E). Subsequently, we carried out MTT, plate cloning, and EdU experiments. The results showed markedly reduced proliferation of EC cells overexpressing ENKUR compared with vector cells (Fig. 2A-C). Flow cytometry assay indicated that cell arrest was exhibited during the G1 phase in ENKUR overexpression EC cells (Fig. 2D, E). Western blotting indicated that in the ENKUR-overexpressing cell line, the protein levels of cyclin D1 (CCND1) and cyclin dependent kinase 4 (CDK4) were decreased (Fig. S1F). We also established a subcutaneous xenograft tumor model in nude mice to further verify the role of ENKUR *in vivo* (Fig. 2F). Xenograft tumors were verified by HE staining (Fig. 2G). IHC study of PCNA expression in xenograft tumors tissues also showed markedly reduced proliferation of ENKUR-overexpressing cells compared with vector cells (Fig. 2G). The tumor volume and weight were significantly lower in the ENKUR overexpression group versus the vector group (Fig. 2H-I).

### Overexpression of ENKUR suppresses the migration, invasion and intrahepatic dissemination ability of EC cells

**Figure 3:**
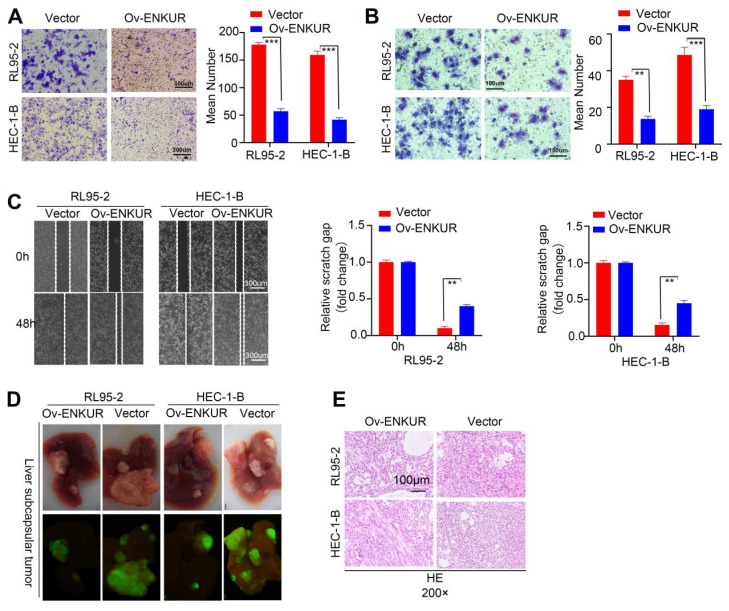
ENKUR decreased the invasion, migration, and intrahepatic dissemination of EC cells. (A) Transwell invasion, (B) Boyden chamber, and (C) cell scratch experiment showed that the capacities of ENKUR-overexpressing cells for migration and invasion were remarkably inhibited. (D) Intrahepatic dissemination model in nude mice, representative liver images for each group (E) HE staining of excised tumor tissues. Student's *t*-test. Data are presented as the mean±standard deviation. **p<0.01, ***p<0.001, ****p<0.0001. Original magnification: 40×. Scale bars: 100 µm, 300 µm. Each bar represents the mean±standard deviation of three independent experiments.

To assess the effect of ENKUR on the migratory and invasive capabilities of cells, Transwell invasion (Fig. 3A), Boyden chamber (Fig. 3B), and cell scratch (Fig. 3C) experiments were conducted. The results showed remarkable inhibition of the capacity of ENKUR-overexpressing cells for migration and invasion. In addition, western blotting showed downregulation of N-cadherin and Vimentin expression and upregulation of E-cadherin in ENKUR-overexpressing cells (Fig. S2A). To further verify the role of ENKUR *in vivo*, we established a intrahepatic dissemination model in nude mice. The numbers of intrahepatic dissemination tumors were calculated and compared. A significantly lower number of nude mice with intrahepatic dissemination was noted in the ENKUR-overexpressing group than the vector group (Fig. 3D). The intrahepatic dissemination tumors were dissected, embedded into wax blocks, and sliced. Subsequently, the sections were processed for HE staining (Fig. 3E). These data indicated that ENKUR inhibits tumor migration, invasion and intrahepatic dissemination in EC.

### Targeting ENKUR restores cell growth, migration, and invasion

**Figure 4:**
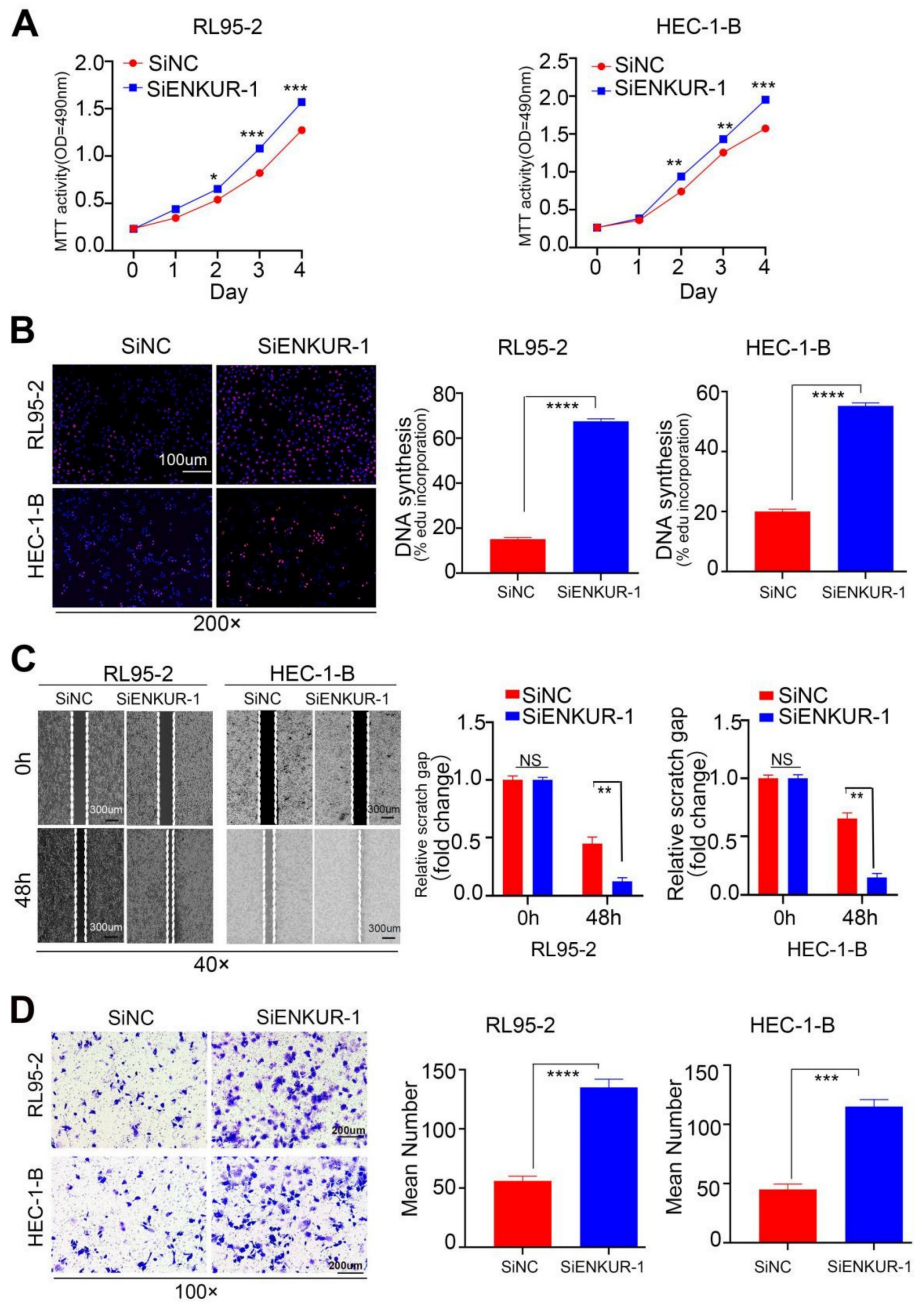
Silencing ENKUR restores cell growth, migration, and invasion. The effects of targeting ENKUR on the cell proliferation of RL95-2 and HEC-1-B cells were examined using (A). MTT assay and (B) EdU experiments. The effects of targeting ENKUR on the migratory and invasive abilities of RL95-2 and HEC-1-B cells were examined using (C) Cell scratch and (D) Transwell invasion experiments. Student's *t*-test. Data are presented as the mean±standard deviation. *p<0.05, **p<0.01, ***p<0.001, ****p<0.0001. Original magnification: 100×, 200×. Scale bars: 100 µm, 300 µm. Each bar represents the mean±standard deviation of three independent experiments.

To further investigate the actions of ENKUR on EC, RL95-2 and HEC-1B cell lines were transfected with ENKUR siRNAs (Fig. S1G). The siRNA fragment #1 was selected for subsequent experiments. Next, MTT (Fig. 4A) and EdU experiments (Fig. 4B) were performed to verify changes in the proliferative ability of cells. The findings showed that targeting ENKUR cells significantly increased the number of proliferating EC cells. Cell scratch (Fig. 4C) and Transwell invasion (Fig. 4D) experiments showed that the migratory and invasive capabilities of targeted ENKUR cells were remarkably inhibited.

### ENKUR combines with CTNNB1

**Figure 5:**
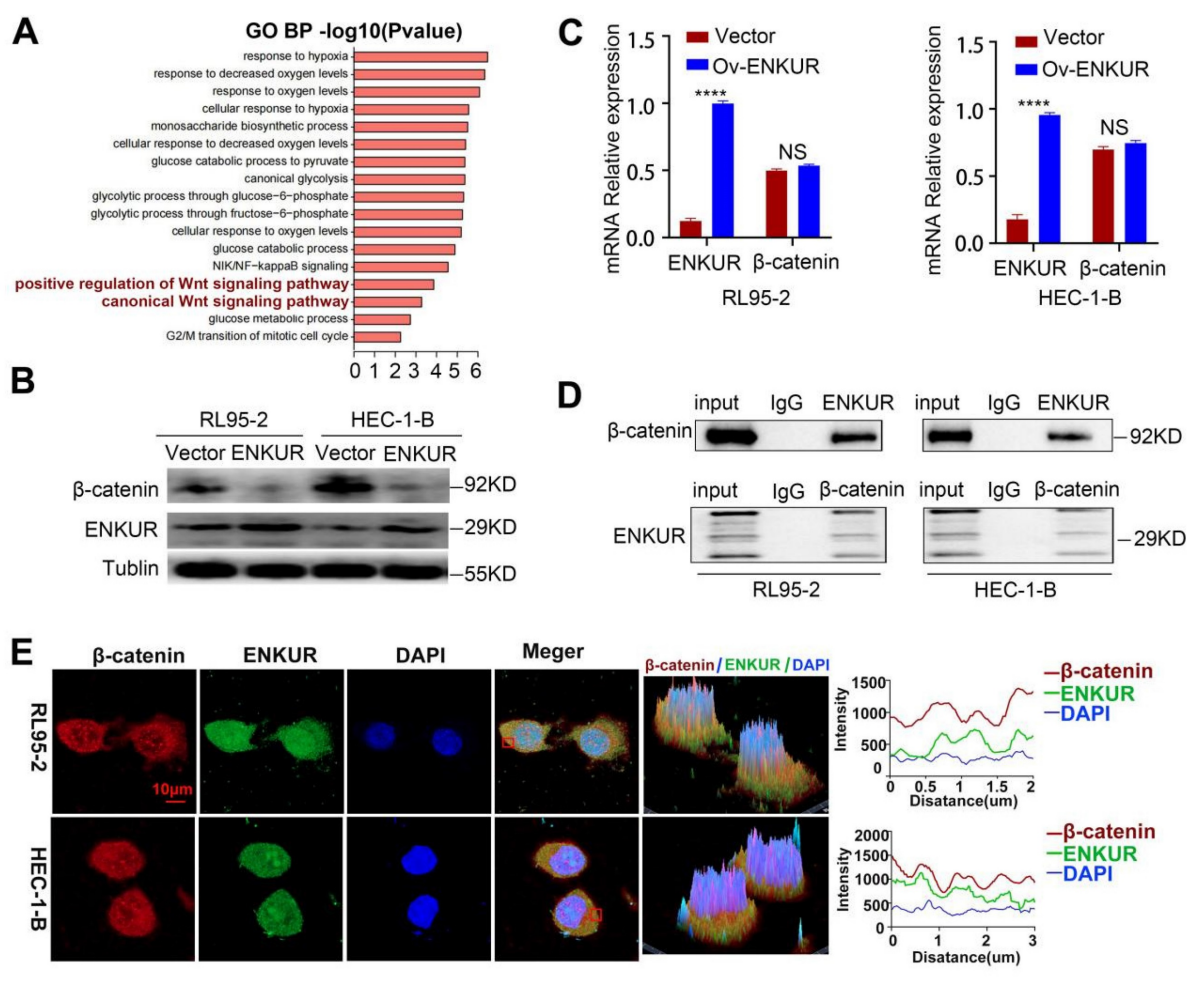
ENKUR interacts with CTNNB1. (A) GO pathway based on the differential interaction protein expression of cell lines (RL95-2, HEC-1-B) with ENKUR or vector group. (B)CTNNB1 were detected in the ENKUR overexpression EC cells by WB experiments, tublin served as a loading control. (C) The transcript levels of CTNNB1 mRNA were measured by RT-PCR. (D) Interactions of ENKUR and CTNNB1 in RL95-2 and HEC-1-B cells were detected by Co-IP analysis. (E)The colocalization of ENKUR and CTNNB1 were visualized by immunofluorescence staining in EC cells. Scale bar, 10 µm.

We attempted to elucidate the inhibition mechanisms of ENKUR in EC. Co-IP of cells with stable ENKUR overexpression and mass spectrometric analysis were performed to identify the partners that interact with ENKUR in EC (Fig. S2B). Furthermore, GO pathway enrichment analysis was performed on differential proteins. The analysis showed that ENKUR was involved in cell response through the canonical Wnt signaling pathway, etc. (Fig. 5A). Western blotting experiments revealed a marked decrease in the expression of canonical Wnt signaling pathway-related protein CTNNB1 in ENKUR-overexpressing EC cells (Fig. 5B). However, there was no significant alteration in* CTNNB1* mRNA expression (Fig. 5C). BioGRID database analysis predicted that CTNNB1 interacts with ENKUR (Fig. S2C). In addition, Co-IP experiments were conducted in RL95-2 and HEC-1-B cells to confirm their interaction (Fig. 5D). Immunofluorescence staining also revealed colocalization of ENKUR with CTNNB1 in the cytoplasm and nucleus of EC cells; of note, cytoplasm localization was more pronounced (Fig. 5E). Overall, these results suggest that ENKUR binds with CTNNB1 in EC cells.

### ENKUR interacts with FBXW7

**Figure 6:**
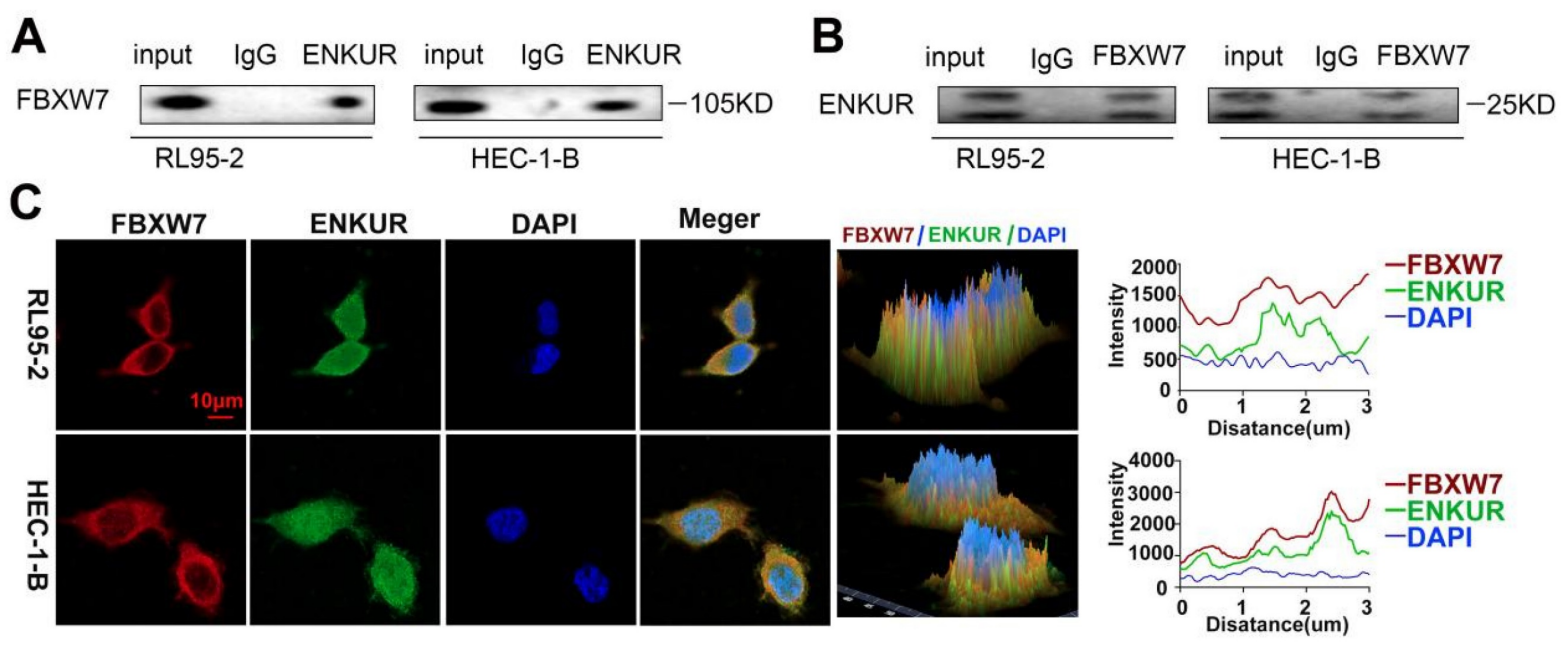
ENKUR interacts with FBXW7. (A and B) Co-IP analysis was performed to assess the interaction of ENKUR and FBXW7 in RL95-2 and HEC-1-B cells. (C) Colocalization of ENKUR and FBXW7 was evaluated by immunofluorescence staining. Scale bar: 10 µm.

We further investigated the regulatory mechanisms of ENKUR in EC. Co-IP experiments were conducted in RL95-2 and HEC-1-B cells to verify the interaction between ENKUR and FBXW7 (Fig. 6A, B). Immunofluorescence staining also revealed colocalization of ENKUR and FBXW7 in the cytoplasm of EC cells (Fig. 6C). Overall, these results suggest that ENKUR interacts with FBXW7 in EC cells.

### FBXW7 interacts with CTNNB1

**Figure 7:**
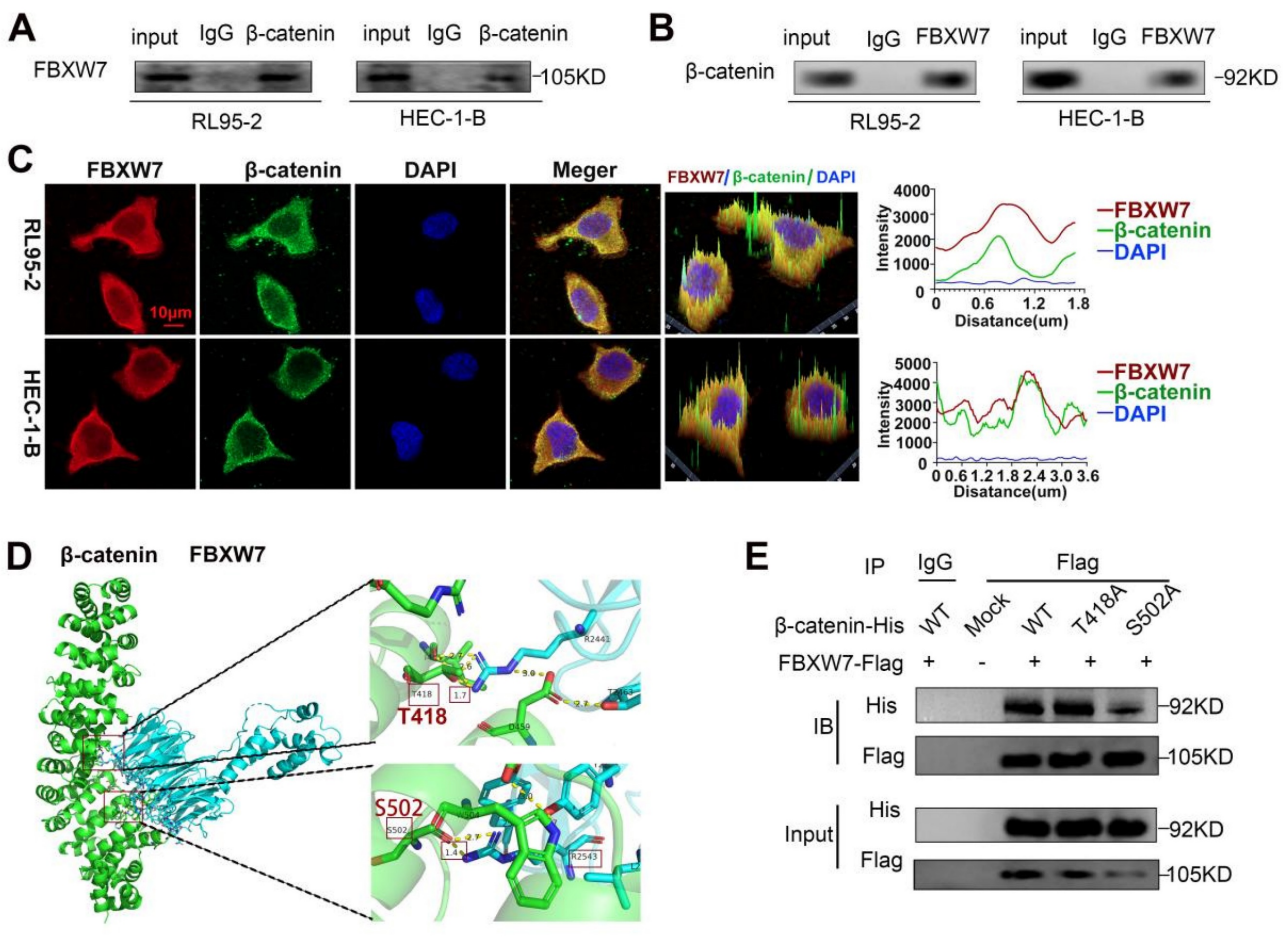
FBXW7 binds to CTNNB1. (A and B) Co-IP analysis was performed to assess the interaction of FBXW7 and CTNNB1 in RL95-2 and HEC-1-B cells. (C) Colocalization of CTNNB1 and FBXW7 was evaluated by immunofluorescence staining. (D) Detailed binding sites between FBXW7 and CTNNB1. Key residues of FBXW7 (cyan) and CTNNB1 (green) are displayed as sticks. Hydrogen bonds are displayed in yellow dash lines and the distances (acceptor to donor heavy atom) of hydrogen bonds are labeled. (E) HEK293T cells were transfected with the mutants of CTNNB1 and analyzed by immunoprecipitation using an anti-Flag antibody. Scale bar: 10 µm.

BioGRID database analysis was conducted to evaluate CTNNB1 as a potential FBXW7-binding protein (Fig. S2D). Co-IP assay and immunofluorescence staining showed cytoplasmic colocalization of CTNNB1 and FBXW7 proteins in EC cells (Fig. 7A-C). Western blotting experiments revealed a marked increase in the expression of CTNNB1 in FBXW7 -deducing EC cells (Fig. S2E-F). To further investigate the potential interaction of CTNNB1 and FBXW7, protein-protein docking was carried out using the HDOCK Server. Hydrogen bonding interactions were formed between the T2421, Y2519, T2463, and D2642 of FBXW7 with R386, E562, D459, and F560 of CTNNB1, respectively (Fig. S2G). In addition, the distances between the acceptor and donor heavy atoms of the two hydrogen bonds were both less than 3 Å, indicating bond stability. We selected the two binding sites with the shortest distance on CTNNB1 (i.e., T418 and S502), which correspond to R2441 and R2543 on FBXW7, respectively (Fig. 7D). We mutated these two sites to alanine residues (T418A, S502A) to test their stability and function. The results of Co-IP assays revealed a significant reduction in the binding affinity between FBXW7 and the CTNNB1 S502 mutant compared with the wild-type (WT) (Fig. 7E). Cells transfected with pCMV-CTNNB1(human)-WT-8×His-Neo served as the CTNNB1-WT group. These results suggested that CTNNB1 mutation at the S502 site has a significant impact on the CTNNB1-FBXW7 protein interaction.

### ENKUR ubiquitinates and degrades CTNNB1 via recruiting FBXW7

**Figure 8:**
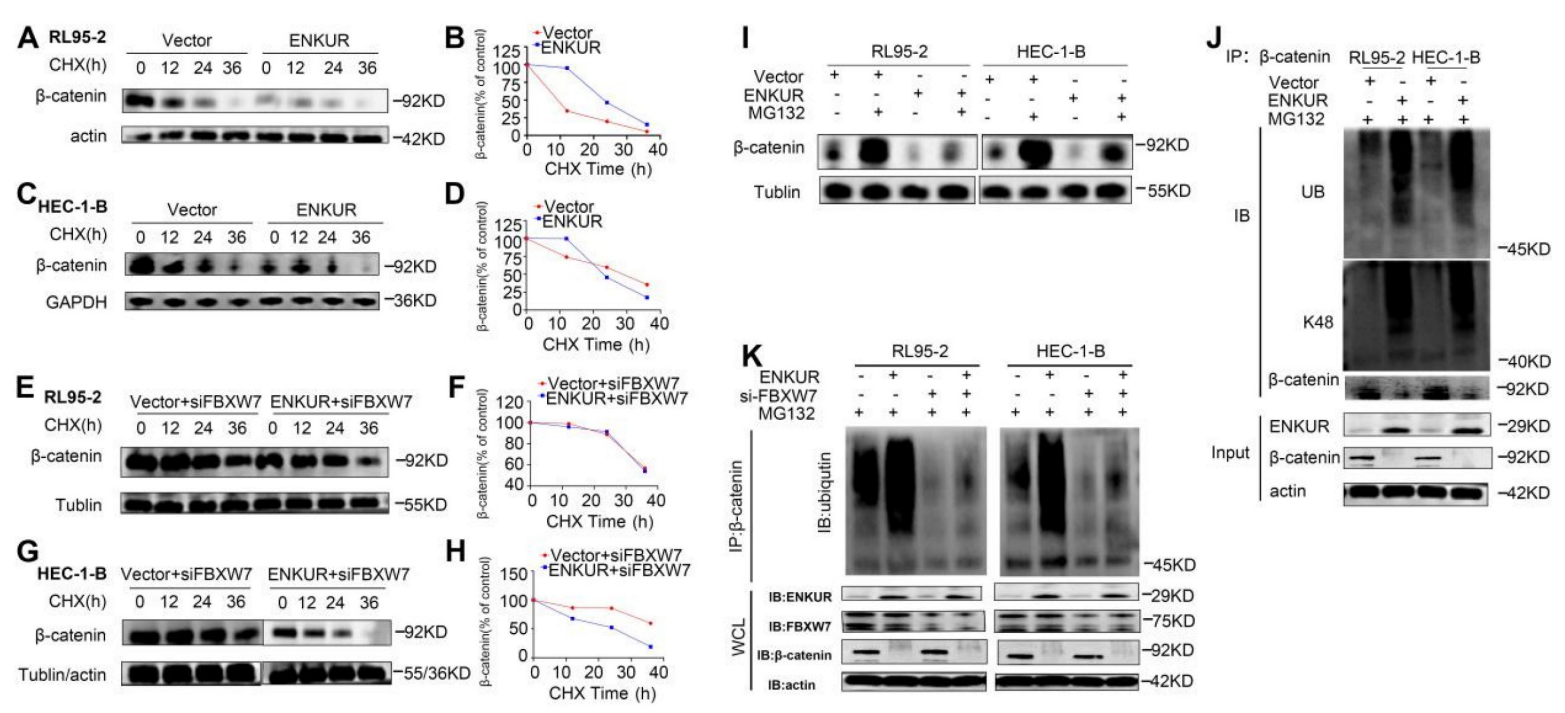
ENKUR accelerates CTNNB1 ubiquitination through FBXW7 recruitment. (A,C) CHX chase analysis of CTNNB1 protein half-life in ENKUR over-expressing and Vector group in RL95-2 and HEC-1-B cells. CHX (50 µg/ml). (E,G) CHX chase analysis detected the effects of FBXW7 knockdown on protein stability of CTNNB1. (B,D,F,H) The half-life curves of CTNNB1 protein in RL95-2 and HEC-1-B cells. (I) The effects of MG132 (20 µM) treatment on the stability of the CTNNB1 protein in the Vector and ENKUR overexpression groups. (J) Co-IP and western blotting assays was detected the effect of ENKUR overexpression on CTNNB1 ubiquitination at K48. (K) Co-IP and western blotting assays were used to examine the effect of ENKUR overexpression and treated with siFBXW7 on the ubiquitination level of β-catenin.

Ubiquitination analysis was performed to further investigate the influence of ENKUR on CTNNB1. CHX chase assay revealed that the CTNNB1 half-life was significantly shorter in ENKUR-overexpressing cells versus vector cells (Fig. 8A, C). Moreover, CHX chase assay results showed that decreased expression of FBXW7 reduced the effect of ENKUR overexpression on CTNNB1 protein levels (Fig. 8E, G). In addition, CTNNB1 downregulation induced by ENKUR overexpression was reversed following treatment with MG132 for 12 h (Fig. 8I). Thereafter, the results of immunoprecipitation and western blotting analyses determined that, depending on FBXW7, the overexpression of ENKUR promoted K48-linked CTNNB1 ubiquitination (Fig. 8J, K). These results suggested that ENKUR mediated the ubiquitination and degradation of K48-linked CTNNB1 through the recruitment of FBXW7.

### FBXW7 ubiquitinates and degrades CTNNB1 at the K281 and K394 sites

**Figure 9:**
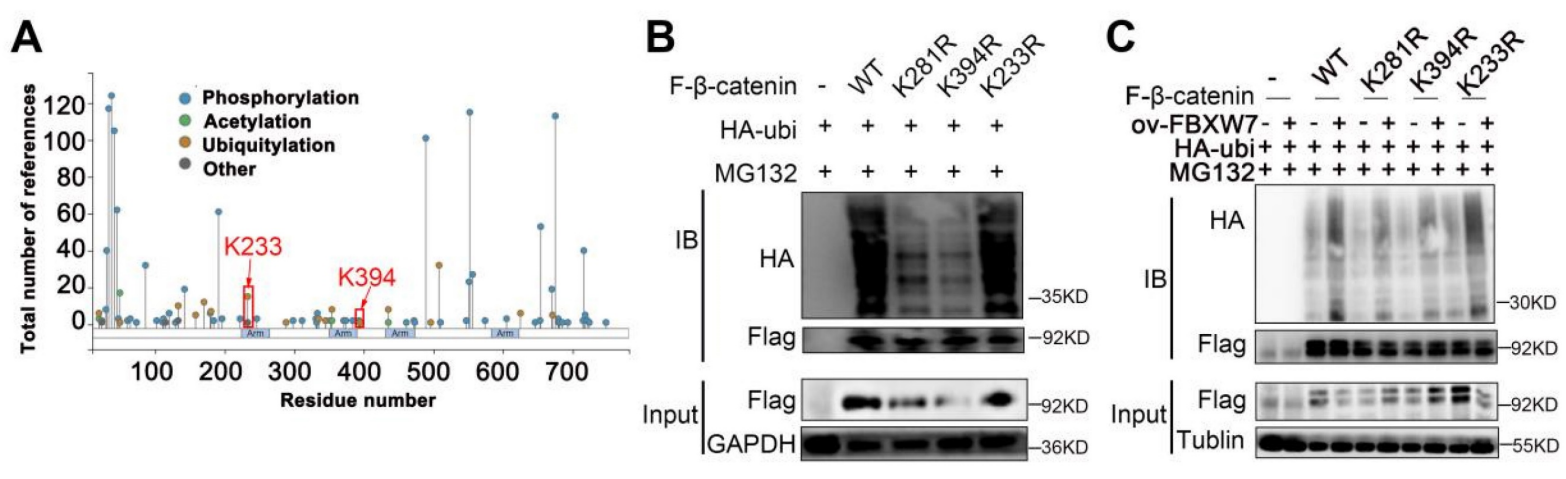
The predicted binding sites between CTNNB1 and FBXW7. (A) PhosphoSitePlus analysis showing potential ubiquitin-modified amino acid sites in CTNNB1. (B) Comparison of ubiquitination modification between wild-type CTNNB1 and CTNNB1 with different mutations. (C) Effect of FBXW7 on CTNNB1 ubiquitination at the K233R, K394R, and K281R sites.

We further investigated the mechanism by which FBXW7 catalyzed the ubiquitination and degradation of CTNNB1. PhosphoSitePlus and GPS-UBER databases were used to search for potential ubiquitin-modified amino acid sites in CTNNB1. The results suggested that K233, K394, and K281 are potential CTNNB1 ubiquitination sites (Fig. 9A and Table 3). Plasmids with mutations of lysine to arginine at the potential ubiquitination sites were constructed to prevent ubiquitination. Next, 293T cells were transfected with plasmids expressing HA-UB, K233R, K394R, and K281R of CTNNB1, and wild-type β-catenin (CTNNB1-WT); cells transfected with pCMV-CTNNB1(human)-WT-3×FLAG-Neo served as the CTNNB1-WT group. The results showed that cells transfected with CTNNB1-WT and CTNNB1 with K233R generated a normal ubiquitination signal, whereas those with mutated K394 and K281 ubiquitination sites underwent remarkably reduced ubiquitination modification (Fig. 9B). Subsequently, we observed that FBXW7 significantly reduced CTNNB1 ubiquitination levels in the K394- and K281-mutant groups compared with the CTNNB1-WT group. These findings suggested that K394 and K281 of CTNNB1 are important for FBXW7-mediated CTNNB1 ubiquitination, and that mutations of these sites can significantly reduce UB modification catalyzed by FBXW7 (Fig. 9C). These results demonstrated that K394 and K281 are key regulatory sites through which FBXW7 mediates CTNNB1 ubiquitination modification.

### Overexpression of CTNNB1 restores ENKUR-induced repression of cell growth, migration, and invasion

**Figure 10:**
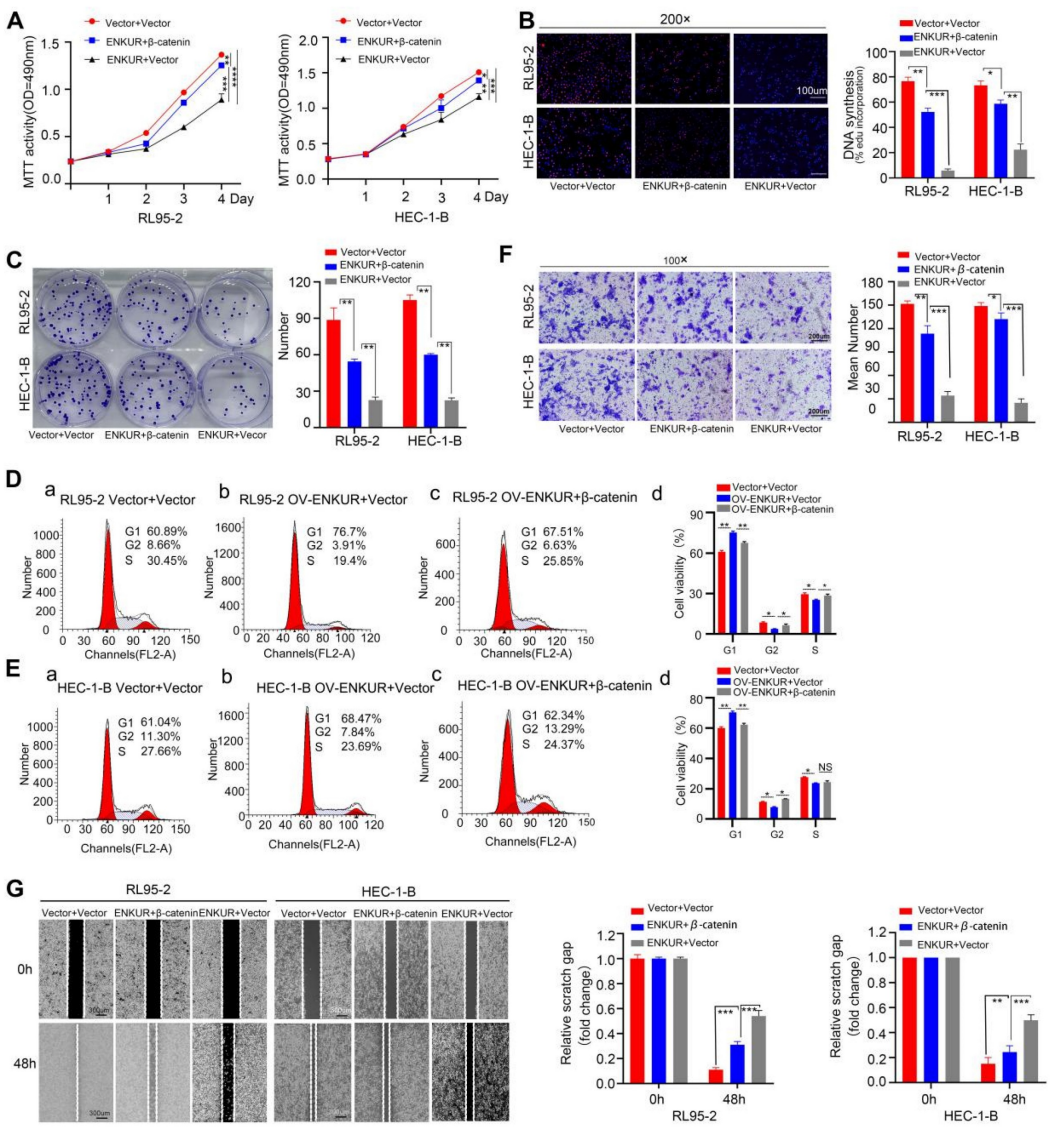
Overexpression of CTNNB1 restores ENKUR-induced repression in cell growth, migration, and invasion. The effects of transfection of RL95-2 and HEC-1-B OV-ENKUR cells with CTNNB1 on cell proliferation were examined by (A) MTT assay, (B) EdU experiments and (C) Colony formation assays. The effects of transfection of RL95-2 and HEC-1-B OV-ENKUR cells with CTNNB1 on the cell cycle were examined by (D, E) Flow cytometry. The effects of transfection of RL95-2 and HEC-1-B OV-ENKUR cells with CTNNB1 on cell migration and invasion were examined by (F) Transwell invasion experiments and (G)Cell scratch. Student's *t*-test. Data are presented as the mean±standard deviation. *p<0.05, **p<0.01, ***p<0.001, ****p<0.0001. Original magnification: 100×, 200×. Scale bars: 100 µm, 300 µm. Each bar represents the mean±standard deviation of three independent experiments.

To confirm the relationship between ENKUR and CTNNB1, CTNNB1 was overexpressed in ENKUR-overexpressing EC lines (ENKUR+CTNNB1 group) by plasmid transfection. MTT analysis, plate clone formation assay, and EdU assay showed that the number of proliferating EC cells was partly restored in ENKUR+CTNNB1 EC (Fig. 10A-C). Flow cytometry findings indicated a restoration in the transition from G1 phase to S phase/G2 phase in ENKUR+CTNNB1 EC (Fig. 10D, E). Similarly, cell scratch and Transwell invasion experiments demonstrated that the cell capacities for migration and invasion were partly restored in ENKUR+CTNNB1 EC (Fig. 10F-J). In addition, western blotting showed upregulation of CTNNB1, N-cadherin and Vimentin expression and downregulation of E-cadherin; however, the ENKUR and FBXW7 levels were not altered (Fig. S2H-I). These data indicated that ENKUR inhibits EC proliferation, growth, and invasion through CTNNB1.

## Discussion

Rapid cell proliferation and metastasis are responsible for the uncontrolled progression of EC. Previous studies have identified numerous factors that regulate EC initiation and development, such as p53, phosphatase and tensin homolog (PTEN), telomere and telomerase-associated proteins, CTNNB1, adipokines, hormone receptor biomarkers (such as estrogen receptor, progesterone receptor and androgen receptor mismatch repair deficient), high microsatellite instability, polymerase epsilon (POLE) exonuclease domain mutated, and other potential biomarkers^7^, ^28^-^39^. Nonetheless, these abnormal changes in genes partly explain the occurrence, development, and prognosis of EC.

Our previous studies demonstrated that the tumor suppressor ENKUR participates in the occurrence, development, and chemosensitity of certain tumors by modulating different genes. These effects include stabilization of p53 expression to suppress NPC metastasis^20^, suppression of myosin heavy chain 9-mediated (MYH9-mediated) c-Myc deubiquitination in LUAD^18^, antagonism of CTNNB1/c-Jun/MYH9/USP7 pathway through an increase in c-Myc ubiquitylation degradation to suppress cell cycle and epithelial-to-mesenchymal transition signals in HCC^19^.

In the present study, we continued to investigate the role of ENKUR in EC. Although ENKUR mRNA and protein expression levels did not show significant differences between EC and normal endometrium tissues, higher ENKUR expression is predictive of a favorable outcome in patients with EC based on the GEPIA and UALCAN database analyses. However, these differences did not reach statistical significance with possible reasons including the detection of EC at an early stage and the high 5-year relative survival rate (>85%) in those undergoing surgical removal of EC^2^, ^40^. Unexpectedly, we observed that ENKUR protein is significantly down-regulated in EC tissues compared with control endometrial epithelial tissues. In addition, we noted that reduced ENKUR expression results in a shorter overall survival time of patients with EC. These results are similar to our previous data in NPC and HCC, as well as those obtained from the GEPIA database^19^, ^20^. Collectively, these findings suggest that ENKUR (as a tumor suppressor) is involved in the pathogenesis of EC.

Subsequently, we investigated the mechanism underlying the effects of ENKUR in EC. Data obtained from a series of experiments showed that ENKUR overexpression in EC cells decreased cell proliferation, migration, invasion, and intrahepatic dissemination, arrested the cell cycle in the G1 phase, suppressed the CCND1, CDK4 and epithelial-to-mesenchymal transition signals. These results further supported the notion that ENKUR may act as tumor suppressor in EC. This is consistent with previous reports on the role of ENKUR in CRC, LUAD, HCC etc.^19^-^21^.

In previous studies, ENKUR-mediated suppression of phosphatidylinositol 3 kinase/protein kinase B (PI3K/AKT) signaling reduced CRC cell proliferation and invasion. In addition, it was reported that ENKUR suppresses LUAD cell proliferation and invasion by directly interacting with PI3K downstream, and may be involved in the PI3K/AKT and mitogen-activated protein kinase/extracellular signal-regulated kinase (MAPK/ERK) signaling pathways^17^. Our previous studies also showed that ENKUR or its enkurin domain binds to FBXW7, CTNNB1 and MYH9, thereby inhibiting carcinogenic factors c-JUN and p53. In turn, this effect leads to the suppression of tumor growth, invasion, migration and metastasis^18^-^20^. In addition, ENKUR could be induced by the small molecule chemical compound cinobufotalin and overcome resistance to cisplatin and sorafenib in NPC and HCC^18^-^20^. However, the molecular mechanism underlying the suppressive effects of ENKUR on the occurrence and progression of EC has not been elucidated.

In the present investigation, we used Co-IP and mass spectrometry analysis to identify proteins that interact with ENKUR; this analysis yielded 285 potential interactive proteins. Subsequently, we conducted GO pathway enrichment analysis for these proteins. We found that ENKUR is significantly involved in the classical Wnt signaling pathway. It is well established that CTNNB1 is an important component in the classical Wnt signaling pathway^41^-^45^. The aberrant activation of the Wnt/CTNNB1 signaling pathway promoted the proliferation, invasion, migration and epithelial-to-mesenchymal transition of EC cells^46^-^48^. Previous studies demonstrated that ENKUR binds to CTNNB1, and suppresses its nuclear transfer through interaction of CTNNB1 with the IQ motif domain in HCC and NPC^19^, ^20^; this process does not induce changes in the total CTNNB1 protein levels. It has been shown that ENKUR binds to CTNNB1 to indirectly decrease its protein levels in LUAD^18^.

Next, we identified that ENKUR interacts with CTNNB1 and colocalized in the cytoplasm and nucleus. Furthermore, we found that the CTNNB1 protein levels were markedly decreased in ENKUR-overexpressing cells, whereas they were increased in ENKUR-knockdown cells. However, there was no significant change in CTNNB1 mRNA levels observed after ENKUR overexpression, indicating that ENKUR might modulate CTNNB1 protein through post-transcriptional modifications. However, the mechanism by which ENKUR regulates the levels of CTNNB1 protein in EC remains unknown.

FBXW7, a member of the SCF E3 ligase family, functions as a tumor suppressor in some tumors via targeting multiple transcriptional activators and proto-oncogenes for UB-mediated degradation^49^, ^50^. Interestingly, the BioGRID analysis predicted that FBXW7 protein interacts with CTNNB1 protein. Our results confirmed that FBXW7 binds to CTNNB1 and colocalizes in the cytoplasm. In addition, CTNNB1 protein levels were up-regulated following the knockdown of FBXW7. These results are in accordance with previously reported observations in studies^51^, ^52^. However, whether ENKUR recruits FBXW7 to directly ubiquitinate and degrade CTNNB1 is currently unknown. Subsequently, the results of molecular docking assay predicted that FBXW7 binds to CTNNB1. Furthermore, we observed a reduction in the binding affinity between CTNNB1 and FBXW7 after mutating the S502 residue. Finally, we confirmed that the ENKUR-mediated ubiquitination and degradation of CTNNB1 is dependent on the recruitment of FBXW7. In addition, the mutation of K394 and K281 sites markedly reduced the levels of CTNNB1 ubiquitination induced by FBXW7.

In summary, our findings demonstrated that ENKUR reduces EC proliferation, migration, invasion, and intrahepatic dissemination by the recruitment of FBXW7 to directly facilitate the ubiquitination and degradation of CTNNB1. The present data are the first to demonstrate that ENKUR is a tumor suppressor in EC, and may be an important target for EC therapy.

## Supplementary Material

Supplementary figures and tables.

## Figures and Tables

**Table 1:** The expression of ENKUR in EC and Normal endometrium

Group	Case (n)		ENKUR expression	P-value*
	Low	High		
EC	178	126 (70.8%)	52(29.2%)	**<0.001**
Normal endometrium	23	3(15%)	20 (85%)	

EC: endometrial cancer *χ^2^-test was applied to access the expression of ENKUR in EC and Normal

**Table 2:** Correlation of ENKUR protein expression with clinical pathological parameters

Parameters	Case	Low expression	High expression	P value
Age (y)				0.077
<54	78	60	18	
≥54	100	66	34	
Hypertension history				0.229
With	57	38	19	
Without	120	88	32	
Hormonal levels				0.230
ER(+)PR(-)	4	2	2	
PR(+)ER(-)	14	7	7	
ER (-) PR(-)	67	50	17	
ER (+) PR(+)	93	67	26	
Clinical stages				0.495
I	150	104	46	
II	20	15	5	
III	8	7	1	
Histopathological grade				0.947
G1	32	23	9	
G2	57	41	16	
G3	89	62	27	

**Table 3:** ID:sp|P35222|CTNB1_HUMAN Catenin beta-1 OS=Homo sapiens OX=9606 GN=CTNNB1 PE=1 SV=1 (from https://gpsuber.biocuckoo.cn/wsresult.php)

Position	Code	Kinase	Peptide	Score	Cutoff
158	K	General	LATRAIPELTKLLNDEDQVVV	0.4163	0.2655
**233**	K	General	HHREGLLAIFKSGGIPALVKM	**0.4839**	0.2655
**281**	K	General	MAVRLAGGLQKMVALLNKTNV	**0.5891**	0.2655
335	K	General	VNIMRTYTYEKLLWTTSRVLK	0.3478	0.2655
345	K	General	KLLWTTSRVLKVLSVCSSNKP	0.278	0.2655
354	K	General	LKVLSVCSSNKPAIVEAGGMQ	0.4002	0.2655
**394**	K	General	TLRNLSDAATKQEGMEGLLGT	**0.6038**	0.2655
666	K	General	AAVLFRMSEDKPQDYKKRLSV	0.3441	0.2655
