# Supplementary material for: FBXW7 Directly Ubiquitinates and Degrades CTNNB1 Mediating the Suppression of ENKUR in Endometrial Cancer
Source: Int J Biol Sci. 2025 Feb 10;21(4):1801–17. doi: 10.7150/ijbs.104067 (PMC11844281; doi:10.7150/ijbs.104067)
Supplement: Supplementary file 1 — Supplementary figures and tables. [file ijbsv21p1801s1.pdf]

TableS 1.A list of Antibodies used for WB,Co-IP,IF and IHC.

antibodies	Cat.No	Company	Species	Dulution
ENKUR	TA804173	Origene	Mouse	1:2000(WB);1:200(IF);1:10(Co-IP) 1:400(IHC)
β-catenin	pAb#51067-2-AP	Proteintech	Rabbit	1:2000(WB);1:200(IF);1:10(Co-IP)
β-catenin	mAb#66379-1-Ig	Proteintech	Mouse	1:1000(WB);1:200(IF);1:10(Co-IP)
FBXW7	pAb 28424-1-AP	Proteintech	Rabbit	1:1000(WB);1:200(IF);1:10(Co-IP)
PCNA	mAb #13110	Cell Sigaling	Rabbit	1:4000(IHC)
GAPDH	pAb AP0063	Bioworld	Rabbit	1:10000(WB)
β-tublin	mAb MB0009	Bioworld	Mouse	1:10000(WB)
His-tag	66005-1-Ig	Proteintech	Mouse	1:5000(WB)
Flag-tag	F1804	Sigma	Mouse	1:1000(WB)
HA-tag	51064-2-AP	Proteintech	Rabbit	1:5000(WB)
K48-linkage Specific Polyubiquitin	mAb #8081	Cell Sigaling	Rabbit	1:1000(WB)
ubiquitin	PAb 10201-2-AP	Proteintech	Rabbit	1:1000(WB)

TableS 2.The primers used in this study

Primers name		Sequence(5'-3')
	Forward	CATCTACACAGTTTGATGCTGCT
CTNNB1	Reverse	GCAGTTTTGTCAGTTCAGGGA
	Forward	CCAGTTCAACCTCCCCCAAT
ENKUR	Reverse	GGGGCCAGACCACATCAAAT
	Forward	CATGGGTGTGAACCATGAGA
GAPDH	Reverse	GTCTTCTGGGTGGCAGTGAT

TableS 3.SiRNA sequences used in experiment

Name of gene	NO.	Target Seq
	1[a]	GGACTAGTTCCAAAGTACA
ENKUR	2	ACACGACATTGGCATAATT
	1	GGAACCCAAAGACCTGCTA
FBXW7	2	GTTAGTGGTTCTGATGACA
	3[b]	GCGTTGTATGCATCTTCAT

[A]:si-ENKUR-1,[b]:si-FBXW7

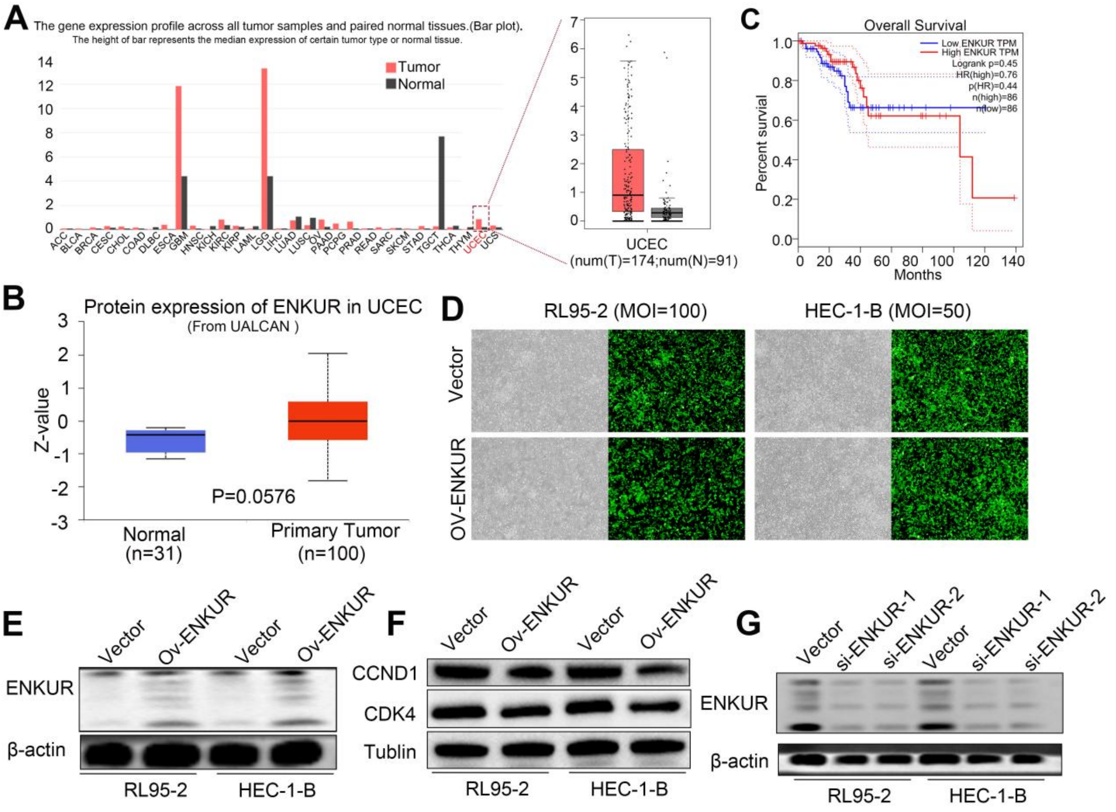

Figure S1. (A)The data from the online database Gene Expression Profiling Interactive Analysis(GEPIA). (B)The data from the online database UALCAN. (C)The affects of ENKUR expression on the overall survival of EC. (D)RL95-2 and HEC-1-B cells were infected with the lentiviral vectors, cells with green fluorescent protein signals. (E)The stable overexpress efficiency of ENKUR was verified by WB.(F)The cell cycle CCND1 and CDK4 protein levels were deceted by WB. (G)The transient knockdown efficiency of ENKUR siRNA was verified by WB.

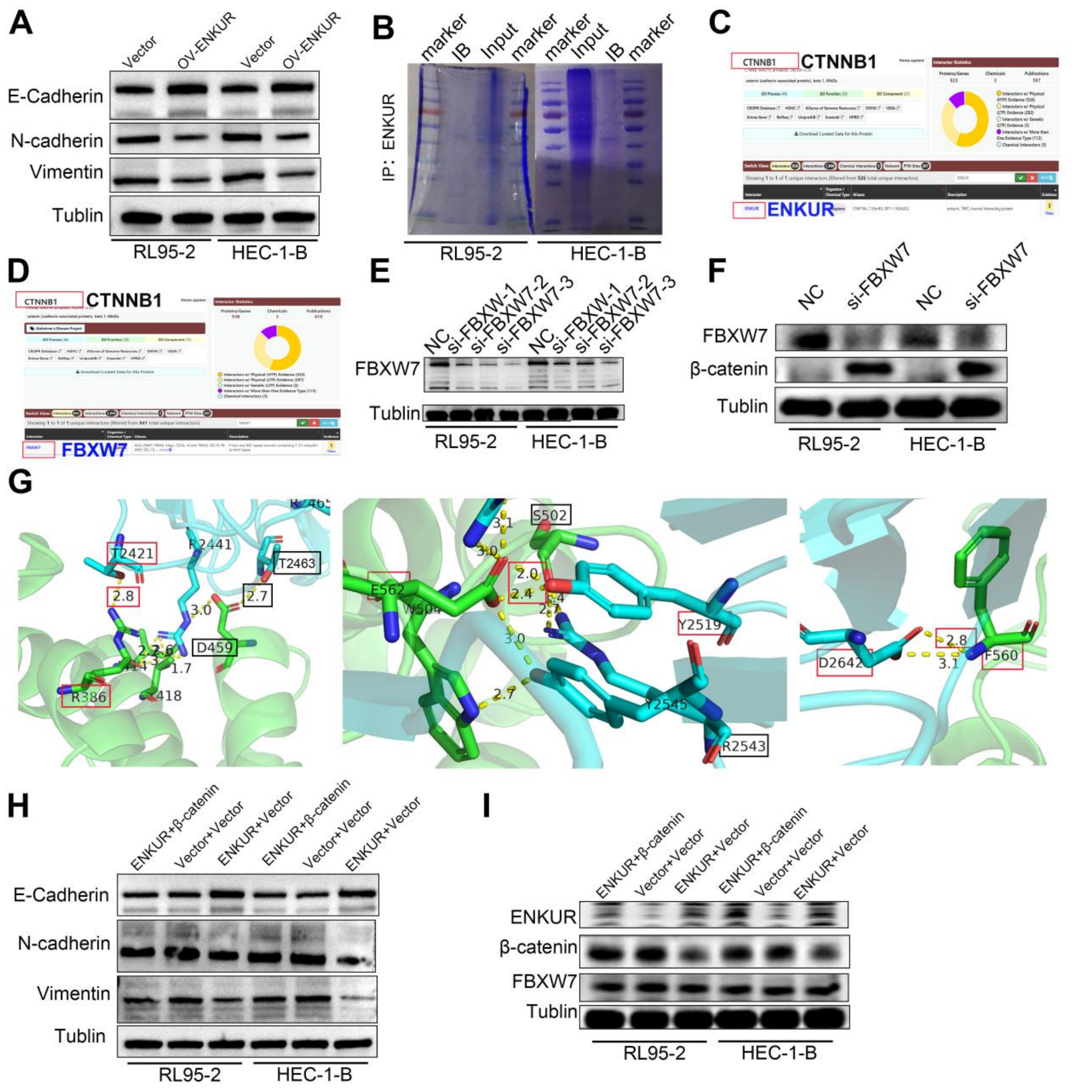

Figure S2. (A)E-cadherin,N-cadherin and Vimentin protein levels were detected in the ENKUR overexpression EC cells by WB experiments, tublin served as a loading control.(B)Immunoprecipitation(IP) was first conducted with anti-ENKUR antibody and stained the CO-IP samples with Coomassie Brilliant Blue after electrophoresis. (C)BioGRID analysis predicts that CTNNB1 protein could interact with ENKUR protein. (D)BioGRID analysis predicts that CTNNB1 protein could interact with FBXW7 protein.(E)The transient knockdown efficiency of FBXW7 siRNA was verified by WB. (F)CTNNB1 and FBXW7 protein levels in FBXW7 transient knockdown EC cells were deceted by WB experiments.(G)Hydrogen bonding interactions were formed between the T2421, Y2519, T2463, and D2642 of FBXW7 with R386, E562, D459, and F560 of CTNNB1, respectively. Key residues of FBXW7 (cyan) and CTNNB1 (green) are displayed as sticks. Hydrogen bonds are displayed in yellow dash lines and the distances (acceptor to donor heavy atom) of hydrogen bonds are labeled.(H)E-cadherin,N-cadherin and Vimentin levels were

detected in the ENKUR+β-catenin EC cells by WB. (I)CTNNB1 and FBXW7 protein levels in ENKUR+β-catenin EC cells were detected by WB.
